# The Effect of Tranexamic Acid Administration on Early Endothelial Damage Following Posterior Lumbar Fusion Surgery

**DOI:** 10.3390/jcm10071415

**Published:** 2021-04-01

**Authors:** Hye Jin Kim, Bora Lee, Byung Ho Lee, So Yeon Kim, Byongnam Jun, Yong Seon Choi

**Affiliations:** 1 Department of Anesthesiology and Pain Medicine, Anesthesia and Pain Research Institute, Yonsei University College of Medicine, Seoul 03722, Korea; jackiedi@yuhs.ac (H.J.K.); DREAMKAIST@yuhs.ac (B.L.); KIMSY326@yuhs.ac (S.Y.K.); BNJUN@yuhs.ac (B.J.); 2 Department of Orthopedic Surgery, Yonsei University College of Medicine, Seoul 03722, Korea; BHLEE96@yuhs.ac

**Keywords:** tranexamic acid, endothelial glycocalyx, spinal surgery, syndecan-1, heparan sulfate

## Abstract

Tranexamic acid (TXA) protects against endothelial glycocalyx injury in vitro. We aimed to evaluate whether TXA could protect against endothelial glycocalyx degradation in patients undergoing posterior lumbar fusion surgery. Patients aged 30–80 years were enrolled. The TXA group was administered a loading dose of 10 mg/kg, followed by a 1 mg/kg/h infusion. Serum syndecan-1 and heparan sulfate concentrations, which are biomarkers of glycocalyx degradation, were measured at preoperative baseline (T0), immediately post-surgery (T1), and 2 h post-surgery (T2). Postoperative complications were assessed, including hypotension, desaturation, and acute kidney injury. Among the 121 patients who completed the study, 60 received TXA. There were no significant differences in the marker concentrations at each time point. However, the postoperative increase in syndecan-1 levels from baseline was significantly attenuated in the TXA group compared with the control group (median (interquartile range); T1 vs. T0: −1.6 (−5.3–2.6) vs. 2.2 (−0.7–4.8), *p* = 0.001; T2 vs. T0: 0.0 (−3.3–5.5) vs. 3.6 (−0.1–9.3), *p* = 0.013). Postoperative complications were significantly associated with the magnitude of the change in syndecan-1 levels (for T2 vs. T0: odds ratio: 1.08, 95% confidence interval: 1.02–1.14, *p* = 0.006). TXA administration was associated with reduced syndecan-1 shedding in patients undergoing posterior lumbar fusion surgery.

## 1. Introduction

Posterior lumbar fusion surgery is a common procedure for the treatment of degenerative spine disease. However, multilevel spinal fusions are associated with substantial blood loss and the increased need for transfusions, and such procedures can be complicated by postoperative morbidity [1]. Surgical trauma and the accompanying inflammatory and immune reactions can result in vascular endothelial injury and the degradation of the glycocalyx [2,3,4]. The endothelial glycocalyx is a gel-like layer composed of glycosaminoglycans, proteoglycans, and glycoproteins covering the endoluminal surface of endothelial cells [4,5]. As an endothelial–vascular barrier, it maintains vascular permeability and oncotic pressure, mediates shear stress, and modulates coagulation and inflammatory responses [4,6,7]. Endothelial glycocalyx damage increases capillary permeability, causes tissue swelling, and evokes abnormal vascular reactions, as well as the worsening of inflammatory and coagulation reactions [7,8]. The association between endothelial glycocalyx damage and morbidity has been observed in critically ill and surgical patients [9,10,11,12]. Therefore, there is an increasing interest to protect endothelial glycocalyx as a promising therapy to reduce morbidity after surgery.

Tranexamic acid (TXA) is a synthetic derivative of lysine that competitively inhibits the activation of plasminogen to plasmin, consequently preventing fibrin degeneration [13]. TXA, as an antifibrinolytic agent, is widely used in trauma resuscitation, as well as in cardiac and orthopedic surgery to reduce blood loss and the need for blood transfusions [1,14]. The protective effects of TXA on endothelial injury have been recently highlighted in vitro [15,16]. In cultured human endothelial cells, the early administration of TXA has been shown to inhibit endothelial glycocalyx degradation caused by ischemia reperfusion injury and sympathoadrenal stress via the inhibition of endothelial sheddase activation [15,16]. However, the potential benefits of early TXA administration in preventing endothelial glycocalyx degradation have yet to be evaluated in a perioperative clinical setting.

The primary purpose of this study was to evaluate whether early administration of TXA could result in endothelial glycocalyx protection in patients undergoing posterior lumbar fusion surgery. We assessed the perioperative changes in the concentrations of biomarkers of glycocalyx degradation, including plasma syndecan-1 and heparan sulfate. As secondary outcomes, we investigated whether intraoperative TXA administration would alter the development of early postoperative complications; additionally, we analyzed the potential associations between the biomarker concentrations and early postoperative complications.

## 2. Materials and Methods

### 2.1. Patient Selection and Study Design

This prospective, randomized, double-blinded study was conducted at Severance Hospital, Yonsei University Health System, Seoul, Korea, in accordance with the tenets of the Declaration of Helsinki. The study protocol was approved by the institutional review board of Severance Hospital, Yonsei University Health System, Seoul, Korea (IRB no. 4-2018-0754, on 26 September 2018) and registered at ClinicalTrials.gov (NCT03697681; first posted 5 October 2018; study start date: 16 October 2018; study completion date: 12 August 2020). This manuscript adheres to the CONSORT (Consolidated Standards of Reporting Trials) 2010 guideline. Data collection started on 16 October 2018 and ended on 31 August 2020. Written informed consent was obtained from all patients prior to study enrolment.

Adult patients aged 30 to 80 years who were scheduled to undergo posterior lumbar fusion surgery from October 2018 to August 2020 were enrolled. Patients were excluded according to the following criteria: allergy and/or hypersensitivity to TXA, current or past history of thrombosis and/or thromboembolism, renal insufficiency (estimated glomerular filtration rate of <60 mL/min/1.73 m^2^), currently receiving treatment with oral contraceptives or anticoagulant medications, and currently being pregnant or lactating. Furthermore, patients who underwent emergency surgery or were unable to read the consent form (for example, due to being illiterate, a foreigner, etc.) were excluded.

After patients were enrolled, an anesthesiologist, who was not involved in either the administration of anesthesia during the surgery or the data collection, performed the group assignments according to a random allocation scheme using a random number generator in Microsoft Excel 2016, with a fixed block size of 4 and a 1:1 ratio. The same anesthesiologist provided two sets of solutions, the experimental drug (10 mL of TXA solution mixed with 90 mL of normal saline) or the placebo (100 mL of normal saline), both of which were colorless and transparent, enabling the double-blinding of the participants, care providers, and those assessing the trial outcomes. The TXA group received intravenous TXA at a dose of 10 mg/kg for 20 min after the induction of anesthesia, followed by a maintenance dose of TXA at 1 mg/kg/h until the end of the operation. An equivalent volume of placebo solution was administered in the same manner in the control group.

All patients underwent standard anesthetic management as practiced in our hospital; briefly, anesthesia was induced with propofol (1–2 mg/kg), remifentanil (4–8 µg/kg/h), and rocuronium (0.6–1.0 mg/kg), and invasive arterial catheterization was performed via the radial artery. Anesthesia was maintained using desflurane (4.0–6.0 vol%) with intravenous remifentanil infusion. During surgery, the mean arterial pressure was maintained at a target of 60–100 mm/Hg. A balanced crystalloid solution (plasma solution A Inj.^®^; CJ HealthCare, Seoul, Korea) was administered in consideration with maintenance requirements, blood loss, urine output, insensible loss, and redistribution. Allogenic packed red blood cells were transfused when a patient’s hematocrit level fell below 24%. If there was acute blood loss exceeding a volume of 500 mL that did not meet the criteria for blood transfusion, Volulyte^®^ (Fresenius Kabi, Bad Homburg, Germany; maximum: 30 mL/kg/24 h) was administered. A significant decrease in mean arterial pressure exceeding 20% was considered to be indicative of hypotension; in such cases, a bolus of ephedrine (4 mg) was administered up to two times. If hypotension persisted, additional doses of ephedrine, phenylephrine, or noradrenaline were administered at the discretion of the attending anesthesiologist, depending on the patient’s vital signs and underlying disease state.

Blood samples were collected for measurements of serum concentrations of syndecan-1 and heparan sulfate at preoperative baseline (T0), at the end of the operation (T1), and at 2 h after the operation (T2). Sera were separated by centrifugation at 1000× *g* for 15 min at 4 °C, then stored at −80 °C until assayed. Commercial enzyme-linked immunosorbent assay kits were used to quantify the serum concentrations of syndecan-1 (Abnova, Taoyuan City, Taiwan, Cat No. KA3851) and heparan sulfate (TSZ ELISA, Waltham, MA, USA, Cat No. HU8718).

### 2.2. Study Endpoints

The primary endpoint was to compare the effects of TXA administration on the perioperative changes in the plasma concentrations of syndecan-1 and heparan sulfate. The secondary endpoint was to investigate the effect of TXA administration on the postoperative complications and the associations between the serum concentrations of syndecan-1 and heparan sulfate and the incidence of postoperative complications during hospitalization. Postoperative complications included postoperative hypotension requiring vasopressor support, desaturation (less than 92% saturation based on pulse oximetry), and acute kidney injury within seven days post-surgery. Hypotension requiring vasopressor support was defined as hypotension that was not corrected by the administration of a 500 mL fluid bolus, requiring an infusion of phenylephrine or noradrenaline. The definition of acute kidney injury was based on the Kidney Disease: Improving Global Outcomes criteria [17].

### 2.3. Sample Size Calculation

No previous study has investigated the changes in the concentrations of syndecan-1 or heparan sulfate in patients undergoing spinal surgery. In a previous study of patients who underwent lung resection surgery, however, the syndecan-1 concentration was 20.2 ± 9.4 ng/mL after 1 h of one-lung ventilation [18]. Assuming that the administration of TXA would induce a change in syndecan-1 concentrations of 25%, the expected mean syndecan-1 concentration was 15.2 ng/mL (rounded up from the second decimal place). Based on this assumption, at least 57 patients were required in each group to be able to detect a difference, with a power of 0.8 and a two-tailed type I error of 0.05. To allow for a possible 10% dropout rate, it was determined that 64 patients were needed in each group.

### 2.4. Statistical Analysis

Data analysis was performed according to the intention-to-treat principal for patients who had measures for all three time points of the paired serum endothelial glycocalyx markers (syndecan-1 and heparan sulfate). In this study, the enrolled patients were divided into two groups (TXA or placebo control groups). Parametric and non-parametric continuous variables (including the degrees of change of syndecan-1 and heparan sulfate concentrations) were analyzed using independent *t*-tests or Mann–Whitney U tests, respectively, based on the results of the Shapiro–Wilk test for normality. The Wilcoxon signed-rank test was used to compare the concentrations of the paired serum markers (syndecan-1 and heparan sulfate) at each time point in each group. Categorical variables were analyzed by the chi-squared test or Fisher’s exact test. Binary data are presented as numbers (%); normally distributed, continuous data are presented as mean ± standard deviation (SD), whereas non-normally distributed, continuous data are presented as medians (interquartile range (IQR)).

Binary logistic regression analysis was performed to assess the associations between the perioperative changes in endothelial glycocalyx marker concentrations and the occurrence of early postoperative complications. The area under the receiver operating characteristic (AUROC) curve was calculated to measure the predictability. The optimal cutoff value was determined by maximization of the Youden index.

All analyses were performed using Statistical Package for the Social Sciences software version 25 (IBM Corp., Armonk, NY, USA), R version 4.0.2 (The R Foundation for Statistical Computing, Vienna, Austria), G*Power version 3.1.9.2 (Franz Faul, Kiel University, Kiel, Germany), and SAS version 9.4 (SAS Inc., Cary, NC, USA).

## 3. Results

### 3.1. Participants and Descriptive Data

**Figure 1:**
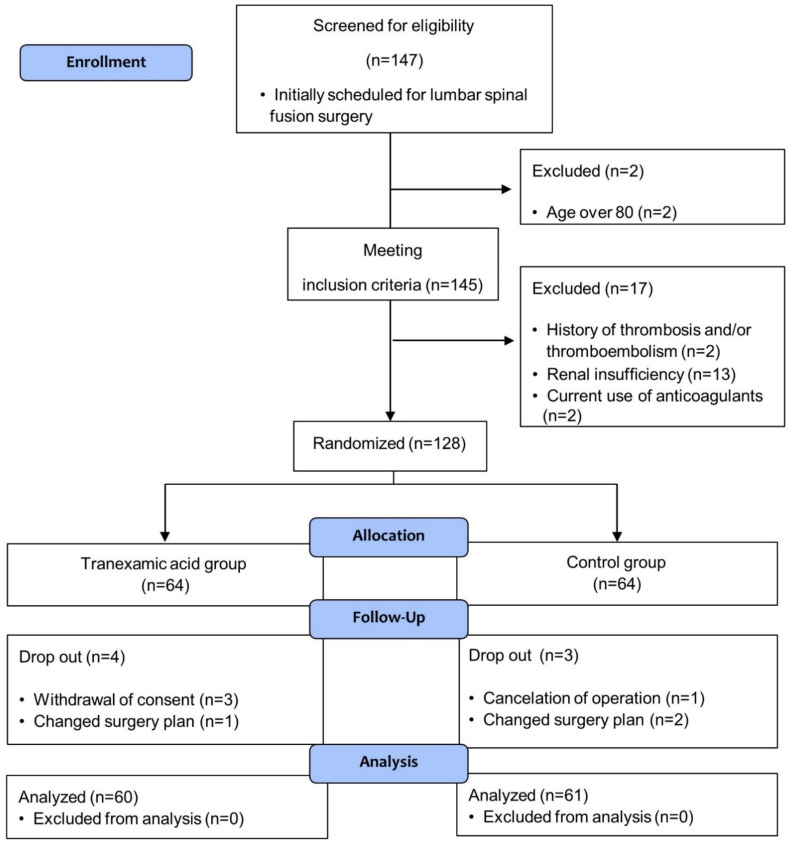
Flow chart of patient enrollment.

Among the 147 patients screened, 128 patients were enrolled; ultimately, 121 patients completed the study with no missing data. Among them, 60 subjects were administered TXA (Figure 1 CONSORT flow diagram). There was no occurrence of side effects following the administration of TXA. There were no statistically significant differences in the preoperative characteristics (Table 1) or the intraoperative measurements (Table 2) between the two groups.

### 3.2. Changes in Plasma Concentrations of Endothelial Glycocalyx Markers

Table 3 shows the baseline and postoperative concentrations of the endothelial glycocalyx markers. There were no statistically significant differences in the marker concentrations at each time point. However, the postoperative increase in the syndecan-1 concentration relative to the baseline level was attenuated in the TXA group compared with the control group. In contrast, there was no difference in the postoperative increase in heparan sulfate concentrations between the TXA and placebo control groups.

### 3.3. Early Postoperative Complications

Hypotension requiring vasopressor support occurred in two patients, acute kidney injury occurred in three, and desaturation occurred in eight. Of those experiencing complications, two complications occurred simultaneously in two patients; thus, early postoperative complications occurred in a total of 11 patients (Table 2). Of these, three patients were in the TXA group and the other eight were in the control group. No statistically significant difference in the incidence of complications was observed between the two groups (5.0% vs. 13.1% in the TXA and placebo control groups, respectively, *p* = 0.121).

We assessed the effects of marker concentrations at each time point and the postoperative changes in marker concentrations relative to the baseline values on the occurrence of early postoperative complications. Only the syndecan-1 concentration at 2 h post-surgery and the change in concentration at that time point relative to the baseline level were significantly related to the occurrence of early postoperative complications (Table 4).

**Figure 2:**
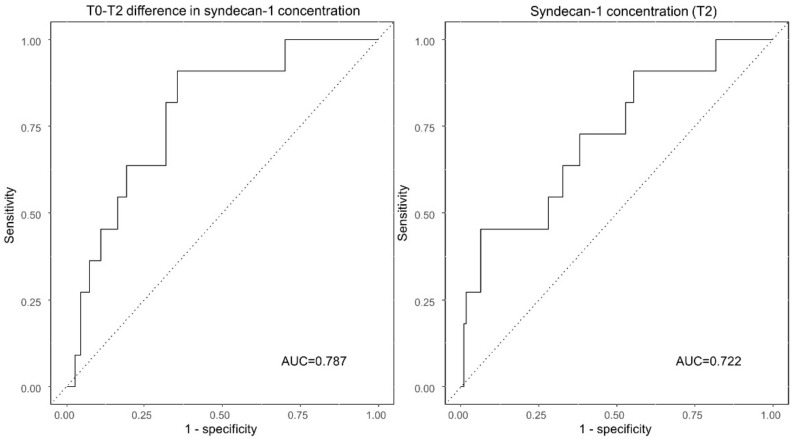
The AUCs for predicting early postoperative complications based on perioperative changes in syndecan-1 concentrations. The AUC for the T0-T2 difference in serum syndecan-1 concentrations is 0.787 (95% CI: 0.663–0.911). In contrast, the AUC for the syndecan-1 concentration at T2 is 0.722 (95% CI: 0.561–0.884). AUC, area under the curve; CI, confidence interval; T0, preoperative baseline; T2, 2 h post-surgery.

Based on the receiver operating characteristic curve analysis (Figure 2), the AUROCs for the curves plotting the ability to predict early postoperative complications based on the changes in syndecan-1 concentrations at 2 h post-surgery (T2 from T0) or the syndecan-1 concentrations themselves at T2 were 0.787 (95% confidence interval (CI): 0.663–0.911, *p* = 0.002) and 0.722 (95% CI: 0.561–0.884, *p* = 0.015), respectively. The optimal cutoff values for predicting early postoperative complication were 3.7 ng/mL (sensitivity, 90.9%; specificity, 64.6%) and 61.3 ng/mL (sensitivity, 45.5%; specificity, 93.6%) for the two curves, respectively.

## 4. Discussion

This randomized controlled study was the first to explore the effects of TXA on the perioperative changes of syndecan-1 and heparan sulfate concentrations in patients undergoing posterior lumbar fusion surgery. Our results showed that the enhanced release of syndecan-1 into the plasma, an indirect indicator of endothelial glycocalyx damage, was attenuated immediately and at 2 h post-surgery compared to preoperative levels in the TXA group. In addition, the magnitude of the increase in syndecan-1 concentrations was correlated with the occurrence of postoperative complications.

The endothelial glycocalyx is mainly composed of proteoglycans, glycoproteins, and glycosaminoglycans (heparan sulfate, chondroitin sulfate, hyaluronic acid, etc.); among these constituents, proteoglycans (syndecan and glypican) and glycoproteins play a role in the formation of the backbone of endothelial glycocalyx. Heparan sulfate and chondroitin sulfate bind to proteoglycans as branch molecules, whereas hyaluronic acid usually binds to the protein CD 44 on endothelial cells [6]. This complex layer serves as a dynamic blood barrier containing several mediators and enzymes, maintaining the osmotic pressure gradient, controlling microcirculatory flow, and regulating anticoagulation and anti-inflammatory reactions [4,19,20]. Oxidative stress, sympathoadrenal activation, tissue trauma, inflammation, and hypervolemia all contribute to endothelial glycocalyx destruction [7,21,22]. Spinal fusion surgery requires a relatively long operation time, often involving a large volume of blood loss with a large wound surface [23]. Therefore, stress and inflammatory responses induced by the surgery itself, in addition to the required administration of a large amount of fluid and blood transfusions, could lead to endothelial glycocalyx damage. Due to the difficulty of performing endothelial glycocalyx imaging in vivo, the damage done to it is mainly inferred from an increase in the concentration of certain blood markers, such as syndecan, hyaluronic acid, or heparan sulfate [21,24]. Lindberg-Larsen et al. showed that methylprednisolone pre-treatment could protect against endothelial glycocalyx damage through the reduction of postoperative plasma syndecan-1 concentrations in a randomized controlled trial (RCT) of 70 patients undergoing total knee arthroplasty [3]. Several other studies have also reported that an increased syndecan-1 concentration in the blood is associated with increased morbidity and mortality, including increased vasopressor use, acute kidney injury, and respiratory failure in patients undergoing surgery or in those with critical illness [6,10,11,12]. These studies suggest that an intervention to facilitate endothelial glycocalyx protection could be a promising therapy to reduce morbidity related to surgery.

The TXA administered inhibits plasmin conversion from plasminogen, thereby reducing fibrin degradation. In addition to the anti-fibrinolytic or clot-stabilizing effects, which are a well-known and primary purpose for using TXA clinically, TXA could theoretically reduce inflammation provoked by plasmin and fibrin degeneration products [13]. This possibility is consistent with the expectation that, independent of its anticoagulation effect, TXA could act by reducing pro-inflammatory cytokine levels in several surgical settings [25,26,27], in addition to the survival benefits demonstrated in trauma resuscitation scenarios in the CRASH-2 trial [28] and MATTERs study [29]. Since inflammation is one of the main insults resulting in endothelial glycocalyx disruption, this appears to be a plausible mechanism through which TXA may exert protection in conjunction with its other established roles. Moreover, in in vitro studies, early intravenous TXA administration protected against endothelial glycocalyx shedding related to oxidative stress and/or sympathoadrenal activation [15,16]. In the aforementioned studies, TXA administration decreased the production of tumor necrosis factor alpha, a pro-inflammatory cytokine, and sheddase/protease activity, both of which are purported to be involved in endothelial glycocalyx disruption. Both anti-inflammatory activity and the inhibition of the enzymatic degradation of endothelial glycocalyx are presumed to be protective. Indeed, our study also provides evidence of the protective effect of TXA; however, the post-surgical increase in heparan sulfate concentrations was not reduced by TXA treatment, implying that the enzymatic inhibition associated with TXA may be discriminative. This dissociative endothelial glycocalyx shedding was also shown in a study by Chappell et al. [24], in which hypervolemia increased serum syndecan-1 and hyaluronic acid levels, but not heparan sulfate levels, implying dissociated degradation. Recently, in a multicenter RCT of 285 patients with moderate to severe traumatic brain injury, patients who received TXA (2 g) within 2 h of trauma exhibited lower syndecan-1 concentrations compared to the control group [30]. Although baseline levels were not measured before the administration of TXA in that study, the results were indicative of an endothelial glycocalyx protective effect of TXA in humans, especially in terms of reducing the release of syndecan-1, which is consistent with our study results.

In spinal surgery, an initial loading dose of 10–20 mg/kg, followed by a maintenance infusion 1–10 mg/kg/h of TXA, is clinically used [31]. In two consecutive in vitro studies using human umbilical vein endothelial cells conducted by Diebel et al., a TXA concentration of 150 µM was protective against endothelial glycocalyx degradation [15,16]. The same authors also showed that both TXA concentrations of 40 µM and 150 µM had protective effects on the glycocalyx in the digestive tract in a previous in vitro study [32]; the 40 µM and 150 µM concentrations are approximately equivalent to the concentrations achieved after administering 10 mg/kg and 30 mg/kg loading doses in vivo, respectively [33]. Therefore, we selected a loading dose of 10 mg/kg, followed by an infusion of 1 mg/kg/h, which is the minimum dose at which clinical effects could be expected while also minimizing the thromboemblic risk caused by TXA administration. Further research is needed to identify the optimal dose to achieve clinically meaningful endothelial glycocalyx protection during various types of major surgeries.

Meanwhile, we investigated the association between the perioperative changes in syndecan-1 and heparan sulfate concentrations and the occurrence of early postoperative complications. As it is known that about five to seven days are required for endothelial glycocalyx recovery following acute degradation in an animal model [34], we selected the time frame for the occurrence of early postoperative complications to be within one week post-surgery. Notably, the postoperative increase in the syndecan-1 concentration was related to the occurrence of postoperative complications, such as hypotension requiring vasopressor support, acute kidney injury, and desaturation. The need for increased vasopressor support to correct hypotension could be explained in connection with a study by Murphy et al. that showed that more vasopressor support was needed as syndecan-1 levels increased [9]. Capillary leakage due to endothelial glycocalyx damage and the consequent decrease in intravascular volume could cause hypotension, leading to the need for more frequent vasopressor use. Moreover, the loss of endothelial glycocalyx integrity and the subsequent inflammation could contribute to acute kidney injury and desaturation. Similarly, in a prospective clinical study of 201 patients with acute decompensated heart failure, high syndecan-1 levels were predictive of acute kidney injury during hospitalization (duration: 8.4 ± 6.5 days) [10]. In addition, in patient groups with sepsis, some studies have demonstrated relationships between higher syndecan-l levels and renal failure (in both pulmonary and non-pulmonary sepsis), acute respiratory distress syndrome (in non-pulmonary sepsis), and a high risk for intubation after large-volume fluid therapy (in cases of severe sepsis) [9,11]. These findings are in agreement with our results; however, in our study, the degree of surgical trauma was not expected to be large, and the occurrence of acute respiratory distress syndrome or respiratory failure requiring mechanical ventilation after posterior lumbar fusion surgery was extremely rare in our center’s experience; therefore, respiratory complications were initially set as desaturation, and all complications resolved within one week after surgery. In contrast, the increase in heparan sulfate concentrations did not show any association with postoperative complications in our study, consistent with the findings of Rahbar et al., who demonstrated that core proteins or constituents, such as syndecan-1 and hyaluronic acid, affect colloid osmotic pressure to a greater degree than do superficial constituents, such as heparan sulfate [35]. In other words, the loss of syndecan-1 would constitute a major disturbance to the system, whereas the loss of heparan sulfate would constitute a minor disturbance [7].

Another notable finding of our study was that the magnitude of the increase in post-surgical syndecan-1 concentrations relative to baselines levels was more predictive of postoperative complications than the concentration itself at any specific time point. As the preoperative baseline status was relatively stable in this study population (with normal kidney function upon elective surgery), an increase in the magnitude of the change seems to better reflect the intraoperative damage that occurs to the endothelial glycocalyx, and it seems reasonable to focus on the extent of this change from baseline rather than the actual concentration at any given time.

This study had several limitations. First, the magnitude of the change in syndecan-1 concentrations was not enormous. Hemodilution and relatively mild surgical stress with a small amount of blood loss might attribute to this; however, despite this fact, a statistically significant difference between the two groups was still shown. We expect this study to form the basis for investigating the protective effects of TXA in surgeries involving greater stress or in situations where endothelial glycocalyx damage is expected to be critical. Second, there was no statistically significant difference in the percentage of patients experiencing early postoperative complications between the TXA and control groups, likely due to the small study population. However, since postoperative endothelial glycocalyx shedding is associated with the occurrence of postoperative complications, and since TXA attenuates endothelial glycocalyx shedding, we expect to draw more clinically meaningful conclusions for the role of TXA through a further large-scale study. Third, plasma markers were used to indirectly infer endothelial glycocalyx damage. Due to the nature of human studies, there were limitations in applying antibody labeling of biomarkers for the immunofluorescence of tissue or direct observation of the endothelial glycocalyx through electron microscopy.

## 5. Conclusions

In conclusion, this study suggested a beneficial effect of early TXA administration in preventing endothelial glycocalyx damage in patients undergoing posterior lumbar fusion surgery, at least in part through the inhibition of syndecan-1 shedding. Although this potential protective effect of TXA did not lead to improved clinical outcomes, further studies are needed to determine the relationship between TXA and endothelial glycocalyx disruption in patients undergoing other types of major surgery.

## Figures and Tables

**Table 1:** Baseline characteristics and intraoperative data.

Variable	Tranexamic Acid *n* = 60	Control *n* = 61
Patient demographic data		
Age (years)	67 ± 8	68 ± 8
Female gender	36 (60.0%)	33 (54.1%)
Height (cm)	159.7 ± 8.5	158.6 ± 8.1
Weight (kg)	65.4 ± 10.6	63.8 ± 10.5
Hypertension	33 (55.0%)	40 (65.6%)
Diabetes mellitus	11 (18.3%)	19 (31.1%)
Cerebrovascular accident	0 (0.0%)	2 (3.3%)
Asthma	1 (1.7%)	3 (4.9%)
Cancer	3 (5.0%)	6 (9.8%)
Preoperative medications		
Beta blocker	5 (8.3%)	8 (13.1%)
Calcium channel blocker	14 (23.3%)	16 (26.2%)
RAS inhibitor	21 (35.0%)	24 (39.3%)
Diuretics	4 (6.7%)	4 (6.6%)
ASA class	2 (2–2)	2 (2–2)
Preoperative Laboratory Data		
Serum C-reactive protein level (mg/L)	1.0 (0.5–2.4)	0.7 (0.4–1.7)
Serum creatinine level (mg/dL)	0.75 ± 0.17	0.80 ± 0.18
Estimated GFR	88.7 ± 11.4	85.0 ± 12.7

Values are mean ± standard deviation, median (interquartile range), or the number of patients (percent). RAS, renin-angiotensin system; ASA, American Society of Anesthesiologists; GFR, glomerular filtration rate.

**Table 2:** Intraoperative data and early postoperative complications.

Variable	Tranexamic Acid *n* = 60	Control *n* = 61	*p*-Value
Intraoperative Data			
Levels fused	2 (1–3)	2 (1–2)	0.605
Duration of anesthesia (min)	240 ± 71	240 ± 68	0.996
Duration of operation (min)	186 ± 65	183 ± 63	0.819
Crystalloids (mL)	1475 (1050–1825)	1400 (1050–1650)	0.773
Colloids (mL)	250 (0–500)	100 (0–500)	0.716
Urine output (ml)	300 (150–535)	260 (150–500)	0.646
Blood loss (mL)	500 (300–800)	500 (300–650)	0.598
Transfused red blood cell (mL)	0 (0–0)	0 (0–0)	0.099
Number of patient requiring vasopressor support *	41 (68.3%)	47 (77.0%)	0.282
Early postoperative complications			
Hypotension requiring vasopressor support	0 (0.0%)	2 (3.3%)	0.496
Desaturation	3 (5.0%)	5 (8.2%)	0.717
Acute kidney injury	0 (0.0%)	3 (4.9%)	0.244
Total †	3 (5.0%)	8 (13.1%)	0.121

Values are mean ± standard deviation, median (interquartile range), or the number of patients (percent). * Number of patients requiring ephedrine bolus more than two times (>8 mg) or phenylephrine/norepinephrine infusion; † total number of patients who developed one or more early postoperative complications.

**Table 3:** Perioperative serum concentration of syndecan-1 and heparan sulfate.

Variable	Tranexamic Acid *n* = 60		Control *n* = 61		*p*-Value †
Syndecan-1 (ng/mL)		*p*-Value *		*p*-Value *	
T0	30.0 (19.6–41.2)		27.3 (19.4–37.1)		
T1	28.4 (18.7–38.0)	0.063	28.8 (19.7–36.9)	0.005	0.617
T2	32.6 (20.1–43.0)	0.324	30.7 (22.1–40.9)	<0.001	0.907
T0-T1 difference	−1.6 (−5.3–2.6)		2.2 (−0.7–4.8)		0.001
T0-T2 difference	0.0 (−3.3–5.5)		3.6 (−0.1–9.3)		0.013
Heparan sulfate (ng/mL)					
T0	1.7 (1.0–2.5)		1.6 (0.9–2.9)		0.762
T1	2.2 (1.3–4.2)	<0.001	1.9 (1.1–3.4)	<0.001	0.544
T2	2.7 (1.8–4.2)	<0.001	2.7 (1.5–4.4)	<0.001	0.893
T0-T1 difference	0.4 (0.1–1.0)		0.2 (0.0–0.9)		0.272
T0-T2 difference	0.8 (0.3–1.8)		0.9 (0.4–1.7)		0.998

Values are median (interquartile range); * *p*-value drawn from Wilcoxon signed-rank test to compare the concentrations of the paired serum markers at T1 and T2 from T0; † *p*-value drawn from Mann–Whitney U tests for comparison between the two groups. T0, preoperative baseline; T1, end of surgery; T2, 2 h post-surgery.

**Table 4:** Results of the logistic regression analysis to assess the associations between perioperative changes in endothelial glycocalyx concentrations and the occurrence of early postoperative outcomes.

	Unadjusted OR (95% CI)	*p*-Value	Adjusted OR * (95% CI)	*p*-Value
Syndecan-1 (ng/mL)				
T0	1.02 (0.99–1.05)	0.128	1.02 (0.99–1.05)	0.139
T1	1.02 (1.00–1.04)	0.102	1.02 (1.00–1.04)	0.093
T2	1.03 (1.00–1.06)	0.024	1.03 (1.00–1.06)	0.023
T0-T1 difference	1.03 (0.99–1.08)	0.147	1.04 (0.99–1.09)	0.123
T0-T2 difference	1.08 (1.02–1.14)	0.006	1.08 (1.03–1.15)	0.005
Heparan sulfate (ng/mL)				
T0	1.09 (0.97–1.21)	0.154	1.10 (0.98–1.23)	0.114
T1	1.08 (0.95–1.22)	0.255	1.09 (0.96–1.24)	0.205
T2	1.10 (0.97–1.25)	0.157	1.11 (0.97–1.27)	0.120
T0-T1 difference	0.65 (0.40–1.10)	0.111	0.62 (0.36–1.07)	0.088
T0-T2 difference	0.94 (0.65–1.34)	0.715	0.93 (0.64–1.35)	0.696

* Adjusted by age and sex. OR, odds ratio; CI, confidence interval; T0, preoperative baseline; T1, end of surgery; T2, 2 h post-surgery.

## Data Availability

The datasets generated for this study are available on request to the corresponding author. The data are not publicly available due to privacy reasons.
